# South African traditional values and beliefs regarding informed consent and limitations of the principle of respect for autonomy in African communities: a cross-cultural qualitative study

**DOI:** 10.1186/s12910-021-00678-4

**Published:** 2021-08-14

**Authors:** Francis Akpa-Inyang, Sylvester C. Chima

**Affiliations:** Programme of Bio & Research Ethics and Medical Law, Nelson R Mandela School of Medicine, and School of Nursing and Public Health, College of Health Sciences, University of KwaZulu-Natal, Durban, South Africa

**Keywords:** Africa, Biomedical research, Communitarianism, Empirical bioethics, Informed consent, Indigenous knowledge systems, Respect for autonomy, *Ubuntu/Botho*, *Ukama*

## Abstract

**Background:**

The Western-European concept of libertarian rights-based autonomy, which advocates respect for individual rights, may conflict with African cultural values and norms. African communitarian ethics focuses on the interests of the collective whole or community, rather than rugged individualism. Hence collective decision-making processes take precedence over individual autonomy or consent. This apparent conflict may impact informed consent practice during biomedical research in African communities and may hinder ethical principlism in African bioethics. This study explored African biomedical researchers' perspectives regarding informed consent and potential limitations to the principle of respect for autonomy in African communities.

**Methods:**

We conducted a qualitative study based on in-depth interviews with 12 biomedical researchers, five females and seven males aged 34 to 74 years, currently working at an African university. Interviews lasted 35–40 min each and involved semi-structured open-ended interviews, which allowed participants to offer information about their perceptions and feelings regarding respect for autonomy and informed consent as practised in Africa. Empirical data from the interviews were recorded, transcribed, and analysed using thematic content analysis, together with an interrogation of relevant scientific literature about African communitarian ethics, making evaluations and drawing inferences consistent with the empirical bioethics approach.

**Results:**

Based on these interviews and analysis of relevant literature, we found that informed consent is difficult to apply in an African context because it derives from a Western conception of libertarian rights-based autonomy. Most respondents pointed out that it was challenging to implement informed consent in the African setting. Furthermore, communalism, customary beliefs, spirituality, and relational autonomy are predominant in most African communities, as exemplified by the African moral philosophies of *Ubuntu/Botho* and *Ukama*, which emphasize communitarianism over individual rights. We also found that language, education, poverty, and cultural beliefs are barriers to obtaining proper informed consent in African communities.

**Conclusions:**

We conclude that there are limitations to applying the principle of respect for autonomy and informed consent in African communities, especially in the context of human biomedical research. We recommend using a more relational approach, such as Ross’s prima facie duties, to implement informed consent in African communities.

**Supplementary Information:**

The online version contains supplementary material available at 10.1186/s12910-021-00678-4.

## Background

Aside from human development, further reductions in mortality rates in human populations require advances in medical technologies, medications, and vaccinations. Further, to achieve such advances, biomedical research has to be undertaken in human populations to determine the safety, efficacy, and cost-effectiveness of these interventions. To provide this evidence base, human participants are required for randomised controlled trials and studies using other designs. Obtaining proper informed consent is crucial to ensuring that we conduct such studies ethically. This work focuses on the comprehensibility of informed consent and its application to the general population of Africa, taking into account that informed consent advocates for individuality. In contrast, most Southern African concepts like *Ubuntu* emphasise communal living. The Khoisan people of South Africa recently developed their code of ethics, motivated by the fact that much biomedical research has been conducted in San communities without obtaining proper consent from the San leaders [1, 2].

The Western-European concept of autonomy, which advocates respect for individual rights [3, 4], may conflict with African cultural values and norms [5–9]. The African worldview advocates for a form of wholeness that comes through one’s relationship and connectedness with other people in the society [6, 7, 9, 10]. The underlying premise is that to be fully human; one must be in a close relationship with others in the community. Since one's existence is dependent on the equal existence of others, 'We exist because of others, and they are because we are' [5, 9, 11]. African communitarian ethics focus on the interests of the collective whole, with this collective typically defined as the family or community, instead of on the individual. Hence, collaborative decision-making processes take precedence over individual autonomy or consent. It is necessary to point out that the community referred to here is not the Western concept: it is the community of humanity, where the individual sees themselves through the viewpoint of others, or as one with the community. In light of this observation, Menkiti [12] opined:

Thus, a crucial distinction between the African view of man and man's idea in Western thought: in the African worldview, it is the community that defines the person as a person, not some isolated static quality of rationality, will, or memory [12].

Such considerations suggest that practitioners of Western biomedical ethics in Africa may encounter intractable ethical problems (moral dilemmas), that might be generated by some of the traditional values, practices, rituals, and taboos that still govern people's behavior and relationships [13]. Following the above argument, it has been noted that moral dilemmas present a challenge to the traditional Western way of ethical reasoning, and may warrant several possible solutions that may seem to be equally valid but mutually exclusive—or that may even seem unresolvable [14]. In any case of ethical disagreement, one needs the ability to recognize and identify the problem and to debate it within a broader framework of agreed-upon rules, established principles, and ethically relevant considerations. Thus, differences in cultural and moral values and seemingly intractable problems within traditional ethical theories may indicate the need to look for a principled alternative approach [15]. This highlights the importance of this study, which explores and suggests possible alternatives to the informed consent process in African communities, using arguments supporting moral pluralism combined with empirical data.

Most commentators have conceived of consent to treatment as a process of shared healthcare decision-making that can encompass the ethical principles of both respect for autonomy and beneficence in the doctor-patient relationship [16–18]. This is demonstrated in the American case of *Grimes v, Kennedy Krieger Institute* (2001) [16, 19], where the Maryland Court of Appeals held that consent could create a contract enforceable by law, if consent agreements contain provisions where "mutual assent, offer, acceptance, and consideration exist". The Court therefore held that researcher/human subject’s consent in non-therapeutic research could create a contract, meaning "a written or spoken agreement intended to be enforceable by law" [16]. In other words, informed consent has been described as the social rules of consent in institutions that must obtain legally valid consent [16, 20, 21]. In its ideal form, the process of obtaining consent from patients or human subjects of biomedical research should consist of a conversation between a healthcare professional (HCP) or researcher and the human subject of biomedical research or healthcare user, which the HCP or biomedical researcher initiates. Such a conversation must encompass transparency, engagement by both parties, and continuity. Such discussions may require evidence that they occurred, such as a witnessed signature or a signed consent form [20]. Such consent agreements may be withdrawn by the human subject at any time. They could also be vitiated by any changes in circumstances that are not communicated to or approved by the human subject of biomedical research or treatment [16, 20–22].

### The African notion of personhood

According to Menkiti [12: 172], in the African worldview the community asserts ontological primacy [12], which means that the individual's reality is secondary and derivative. He argued that "as far as Africans are concerned, the reality of the communal world takes precedence over the reality of the individual life histories, whatever these may be" [12]. Although there are many diverse African cultures, there are many commonalities in value systems, beliefs, and practices [9, 23], which largely reflect the African worldview [5, 9, 23–27]. This worldview is greatly influenced by the African communitarian way of life or ethos [28]. Specifically, Munyaka and Motlhabi argue that this worldview's most abiding principle is *Ubuntu* [23, 29]. We will explore this and other values contributing to tackling the ethical conflict that arises from applying the Western notion of bioethics to biomedical research in Africa.

Many issues surround the relationship between doctors and patients or researchers and research participants in Africa [30]. These may originate because the ethical principles applied in biomedical research are rooted in Western-European moral traditions that emphasise individual autonomy [3, 4, 18, 31]. In contrast, African concepts such as *Ubuntu* [32] and *Ukama* [10], and many more, advocate for a form of wholeness that comes from one's relatedness and connectedness with others in society [12, 31, 33]. The critical concept in Africa is communitarianism, based on the belief that community relationships meld a person's social identity and personality, with minor emphasis placed on the individual [9–12, 23, 31–33]. From the analogy above, it is clear that the African notion of personhood is relational. Other ethical theories, such as the ethics of care and feminist ethics, speak of relational processes [31]. However, this study adopted Ross's model of moral pluralism (prima facie duties) because it is based on responsibilities that we owe to one another in ethical decision-making.

### Ross's model of moral pluralism

The central notion of Ross's alternative approach is *prima facie duties*, which means duties based on the first impression, which are accepted as correct until proven otherwise [34], and are different from absolute obligations held in all circumstances, or conditional duties [3]. Ross argued his position as follows:

Prima facie suggests in this approach that one speaks only of which moral situation presents at first sight and which may turn out to be illusory. Whereas, what he is speaking of is an objective fact involved like the situation or more strictly in an element of its nature, though not as duty proper does, arising from its whole nature [35].

For example, is it one's duty to keep a promise, when one may be in a position to avert a severe accident by failing to keep it? [36]. There are two prima facie duties in this case: the first is the duty to keep a promise, and the second is to relieve distress. The circumstances in a particular case can make the latter a more significant duty when looking at the seven prima facie duties, as Ross [34, 35] postulated, which serve as moral decision-making guidelines. These prima facie duties are fidelity, reparation, gratitude, justice, self-improvement, non-maleficence, and beneficence. One may prefer Ross's model for this research endeavour, because it advocates for moral pluralism—which is the guiding framework for the argument in the paper—as justified and elaborated on below in choosing a conceptual framework for the study. Secondly, the prima facie duties stem from relationships, which appears similar to the African moral philosophy of *Ubuntu* [23, 37, 38]. Although Ross's model is Western in origin, it gives credit to relationships, obligations, and responsibility, similarly to the African notion of *Ubuntu* which advocates for just relationships [23]. This is also similar to other concepts outlined in Ross's prima facie duties: for example, obligations arising from one's actions, such as (a) fidelity (keeping promises) and (b) reparation for wrongful acts, as well as those arising from others' actions, including gratitude for others' kindness and reciprocating generosity. In addition to, the duty of promoting justice, beneficence, and responsibility for self-improvement and improving others' conditions in society, as well as commitment to non-maleficence [34, 35]. It is also important to note that Ross's model is not limited to the micro-sociological level but calls for issues such as reparations, which can translate into restorative and distributive justice at the societal, legal, and policy levels, as argued by some authors and legal authorities [23, 38–40]. This study aimed to explore and suggest possible alternatives to the informed consent process in African communities, using arguments supporting moral pluralism combined with empirical data.

## Methods

### Study rationale

This study involved identifying the contrast between informed consent derived from the principle of respect for autonomy, and African traditional values and belief systems. Specifically, this study involved analysing the application of informed consent in biomedical research in Southern Africa, which will help biomedical researchers and other scholars to understand and determine the impact of culture on such research in Africa. This is based on the premise that rules and norms of informed consent in bioethics are predominantly derived from Western-European intellectual and moral traditions, where the primary emphasis is on respect for individual autonomy.

### Aims and objective

This study's primary objective was to explore the perceptions of biomedical researchers at an African university regarding the comprehensibility of the informed consent doctrine and its application to the general population of Africa. The study's rationale was that informed consent advocates individuality, while most Southern African concepts, such as *Ubuntu* or *Ukama,* emphasise communitarianism [10, 23, 29, 32, 37, 38]. For example, the San peoples of Southern Africa recently developed their code of ethics [1], motivated by the fact that many researchers conducted research studies without obtaining proper consent from the San leaders or community elders [2]. This study was therefore designed to explore the concept of informed consent in the doctor-patient and researcher/human subject relationship, within the context of traditional African values, based on the understanding that Africa's communitarian and other traditional belief systems undoubtedly deepen one's connectedness with family, language, belief systems, and customs, including ancestral worship and spirituality [5, 28, 31, 41, 42]. Thus, there might be conflicts when applying Western-derived principles or concepts of bioethics to indigenous African population groups.

### Choosing a conceptual framework

#### A top-down/deductive or bottom-up/inductive approach?

According to Beauchamp and Childress and others, one can view moral judgements and decision-making in two ways: the 'top-down’ or the 'bottom-up' approach [3, 43–46]. The top-down approach is based on the justifiability of a particular action by applying various moral theories and principles. In contrast, a bottom-up approach is a form of justification that begins with concrete and unmistakable instances of good and bad behaviour, and proceeds to formulate general principles that capture and distil our fundamental moral responses to the case in question [3, 43]. Thus, the traditional ethical theory is a conceptual system that attempts to define and guide the best decisions and actions. It investigates the best way for people to live and what actions are right or wrong in any particular circumstance. The traditional ethical reasoning model seeks to resolve human morality issues by putting in place concepts that define ideologies, like good and evil, right, and wrong, virtue and vice, justice, and crime. Moreover, principles and theories are set out to be followed while making traditional moral judgements [43–46].

Traditional top-down models of reasoning are considered monistic and reductionist, because they believe that all moral considerations could eventually be grounded in one ultimate principle, in terms of normative foundations that can provide solutions to all moral controversies [3, 45, 46]. Beauchamp and Childress argue that traditional ethical theories are reductionist by nature [3], which means they reduce the essence of morality to one all-embracing principle and a single unifying standard of ethics or right action because moral reality is too complicated in itself. The complexity of our decision-making is aggravated even more by non-moral conditions. There is no guideline on choosing the right approach or determining the proper moral and non-moral reasons that justify our actions. The inability to give guidelines justifying our actions establishes that the traditional ethical theory cannot resolve the relationship between the universal and the particular. It cannot clearly state how to apply the general universal principle to a specific case, and this may be why the top-down approach fails [3, 46]. Beauchamp and Childress also argue that traditional moral theories have internal problems that are not helpful in resolving ethical dilemmas and practical issues [3].

Considering Beauchamp and Childress's and others’ critiques of the traditional top-down approach [3, 46], we have instead chosen the bottom-up approach [44, 45], because the conventional moral theories (as demonstrated above) appeal to an abstract universal notion of personhood and could be a means of intellectual and cultural imperialism. Bioethics, defined as 'the application of ethics to all life' [47–49], deals with real people in particular situations, or specific persons in specific contexts or contextualised persons. Furthermore, global bioethics is also rooted in culture. There may be no bioethics from a worldwide and cross-cultural perspective if there is exclusion of cultural practices and ways of life [6–8, 13, 16, 28, 49, 50].

According to Beauchamp and Childress, the four globally accepted ethical principles are respect for autonomy, beneficence, non-maleficence, and distributive justice, postulated to address bioethical issues from a Western-European moral perspective [3]. However, the Western-European worldview is different from the African worldview [4, 5, 9, 23–27]. Thus, one can argue that constructively, in the real sense, bioethics cannot function in the absence of culture, because its elements and principles are based on culture and traditions [13]. Therefore, the case study approach seems most suitable, instead of imposing a priori values and principles. This gives us the ability to distil some answers to pertinent questions from historically, culturally, and otherwise situated cases [51]. The present study assumes that Africa-situated issues should distil some answers to relevant questions regarding local applications of the doctrine of informed consent from the viewpoint of African values, belief systems, and perspectives.

Furthermore, this research study opted out of the monistic approach and chose a pluralistic one because of its compatibility with the idea of multiculturalism [50, 52]. Kevin and Wildes argue that culture has a deep relationship with morality [51]; thus, the concept of multiculturalism links directly to why morality should be pluralistic and diverse. These authors assert that morality encompasses ethical practice, which is embedded in culture, which we see as a way of life [51]. Thus, bioethics seeks to examine those practices and moral systems which are embedded in cultural practices, hence advocating for a multicultural society which could also be synonymous with supporting a morally pluralistic society [16, 50–52].

### Research question

The research question in this study is as follows: Is it possible to find a way to implement the principle of informed consent in the context of African bioethics without undermining traditional values and belief systems?

### Research design

We adopted a qualitative method by utilising semi-structured, in-depth interviews with relevant stakeholders. Qualitative methods are applicable when variables involved in a research study are not controlled [53, 54], and inquiries are taken beyond what happens, to how and why it happens. These pointers justify using a qualitative method for this study, which involved understanding the nature of informed consent and its implications in Africa. A qualitative research approach also allows for an open-ended view of the themes studied, to better understand and explain the phenomena under discussion. This study relied on qualitative empirical data and an extensive review of relevant literature [55], consistent with the recommended integration of empirical data and normative analysis required for standards of practice in empirical bioethics research [57, 58]. The data from these different but equally relevant sources enabled the researchers to draw reasonable inferences and conclusions, and to make suggestions to address the research problem and questions [57, 58].

### Sample population and data collection

The researchers applied semi-structured in-depth interviews to collect data and information concerning applying bioethics principles to the general population of South Africa, and the possibility of reconciling potential conflicts that may arise in their application to traditional African communities. The process required identification of appropriate research participants, and biomedical researchers and practitioners were purposefully identified as the target population. A target number of 12 participants was considered adequate for this study, based on biostatistical consultations and literature review [53–55]. In-depth interviews were subsequently administered to each participant using an interview guide with previously delineated questions, as shown in Additional file 1. The semi-structured and open-ended questions used in the interview allowed participants to offer information about their perceptions, attitudes, and feelings regarding informed consent as practised in Southern Africa. In-depth interviews for this study lasted 30–40 min each and were conducted by a single researcher (FA-I), who served as principal investigator (PI). The PI interviewed the 12 biomedical researchers at their workplaces at the University of KwaZulu-Natal (UKZN). The interviews were recorded using an audio recorder, then transcribed. The data were analysed and combined with results of an extensive literature review in the public domain [55]. Three other invited potential participants were unable to take part due to work commitments and time constraints. However, their absence did not materially impact this study's results, since we achieved the target sample of 12 and data saturation; by the time the PI reached the twelfth interview, we were generating no new data and no new themes were being coded. The researcher obtained rich qualitative data from knowledgeable respondents, in a manner which we believe was replicable, until we could generate no new information from additional data coding [56]. Ultimately 12 respondents were interviewed and completed the study. The participants' characteristics are as shown in Table 1.

**Table 1:** Demographic characteristics of the study participants

	Age (years)	Race	Gender	Field
Participant 1	64	White	Female	Clinical Research Laboratory
Participant 2	38	Indian	Female	Clinical Trials
Participant 3	72	Indian	Female	Pediatric Nephrology
Participant 4	53	African	Female	Chair BREC
Participant 5	46	African	Female	Medical Technologist in Clinical Pathology
Participant 6	39	African	Male	Gynaecologist
Participant 7	57	White	Male	Medical Researcher
Participant 8	56	African	Male	Medical ethics, informed consent, and Traditional Medicine
Participant 9	37	African	Male	General Practitioner
Participant 10	65	White	Male	Bioethics Committee
Participant 11	34	Coloured^a^	Male	Medical Law
Participant 12	74	White	Male	Medical Law

^a^The term ‘Coloured/s’ is a non-derogatory term used to describe a multiracial ethnic group native to Southern Africa, with ancestry from more than one of the various populations inhabiting the region, including Khoisan, Bantu, White, Austronesian, East Asian, or South Asian. https://en.wikipedia.org/wiki/Coloureds [62]

### Sampling methodology

The study initially adopted a random sampling technique for choosing participants. However, the researchers discovered that this technique was ineffective, since the study involved speaking to researchers, lecturers, and HCPs who already have a busy schedule. We therefore decided to adopt the snowball sampling method; this may be described as a technique for finding research subjects where one subject gives the researcher the name of another potential subject, who in turn provides the name of a third, and so on [54]. Snowball sampling can be placed within a broader set of link-tracing methodologies [54, 55], which seek to take advantage of identified respondents' social networks to provide an ever-expanding group of potential contacts [53–55]. This process assumes that a ‘bond’ or ‘link’ exists between the initial sample and others in the same target population, allowing for a series of referrals to be made within a circle of acquaintances [53–55]. The participants in this research study were biomedical researchers at UKZN in South Africa, who had experience of implementing the informed consent process in Africa.

### Data analysis

This study adopted thematic content analysis of the available data to make some evaluations and draw inferences. Thematic content analysis is appropriate for a descriptive presentation of qualitative data [53, 54], and Anderson further suggests that it can be used when the study involves identifying, examining and reporting patterns or themes within a data set [59]. The PI adopted a manual thematic content analysis process, where key terms were sourced and coded from the data using several iterative steps. This iterative approach helped to develop further and modify the coding system and determine when we reached data saturation. First, we read the entire transcript to obtain an overall sense of the data. To describe, interpret and critically analyse the data, the text was summarised using codes, providing a code report. After the initial coding of long texts of verbatim data, expressions with a similar meaning and an immediate part of the context and reference (participant's identifying code) were compiled into categories by classifying and integrating the coded units of data [60]. We further analysed the qualitative categories to identify repetition and possible relations or patterns in the data and compiled them into overarching themes. Conclusions from this analysis were documented in the final descriptive summary, presenting themes and main points from the code report, with verbatim quotations from informants to illustrate them. The PI conducted the primary analysis and coding and a research supervisor cross-checked it, and there were no disagreements regarding the coding and themes generated from the data, as previously reported [55]. The interviewer (PI) was unknown to most respondents when data collection occurred and maintained neutrality during data collection and analysis to minimise researcher bias. Furthermore, this study has been reported in keeping with the Consolidated Criteria for Reporting Qualitative Research (COREQ) [61]. See checklist (Additional file 2).

### Ethical approvals

This study was approved by the Humanities Research Ethics Committee (HREC) at UKZN. All participants were contacted via electronic mail and provided written informed consent before participation in the study, after full information disclosure (Additional file 3). Confidentiality was maintained by safe storage of the data, which were reported anonymously. This article is partly derived from a study conducted in partial fulfilment of the PI’s Master of Population Studies degree attained in 2017 [55].

## Results

### Characteristics of participants

The sample population (n = 12) comprised five females and seven males aged between 34 and 74 years. The sample included four professors, six academic doctors, and two practising medical doctors (clinicians). The demographic characteristics of the research participants are summarised in Table 1.

### Findings from the thematic analysis

Five themes and several subthemes were derived from the interview data: (i) the perception of informed consent by the participants, (ii) the perception of informed consent in Africa, (iii) the participants' perception regarding applying the principle of respect for autonomy and informed consent, (iv) the impact of education on the informed consent process, and (v) the impact of poverty on the informed consent process in Africa. These themes will be presented below. Table 2 shows the themes and subthemes derived from the interview data.

**Table 2:** Main themes and subthemes derived from thematic analysis

Main themes	Subthemes
Perception of informed consent	General perception of participants of informed consent, the process of consenting to research/treatment, the regulations of South Africa on informed consent
Perception of informed consent in Africa	Application of informed consent in African communities, challenges in the application of informed consent in African communities
Perception of the application of the principle of respect for autonomy	The status of applying the principle of autonomy, the challenges in applying the principle of autonomy, the conflict between individual versus community decision making
Perspective of the impact of education on the informed consent process	Lack of Western education, the ability to comprehend the informed consent process, and the ability to make an informed decision
Perspective of the impact of poverty on the informed consent process	Lack of proper financial structure to get access to Western education, vulnerability, therapeutic misconception

Table 3 shows the codes assigned to each participant in the study to maintain confidentiality.

**Table 3:** Code names for research participants

Codes	Interpretation
F	Female
M	Male
R	Respondent
Number	Age of the respondent

For instance, RM74 will refer to a respondent who is male and aged 74

### Respondents' perception of informed consent

Informed consent is a process by which a research participant or patient consents to participate in biomedical research or medical treatment. It is supposed to be undertaken without undue influence and with the human subjects’ volition. This process has stages, as RM56 outlined:

There are crucial elements that you need to have for informed consent to be considered valid. One of the most important things is that informed consent is not just about signing a consent document. Informed consent involves full information disclosure; further, it must ensure full comprehension of information disclosed by the research participants or healthcare users. Permission must be voluntary; that is, the person must be free to agree or disagree to participate in the research. When we talk about voluntary, there has to be the absence of coercion or undue influence. The participants must not join out of fear or any other reason to participate when they are not freely deciding to do so. There must not be an undue inducement. You must not induce them with money or any other thing that could influence the participant into accepting to participate. Finally, there must be justice; you know, individuals, if they have to spend their time and effort to get involved in research, they have to be compensated. If they happen to drop out of the research study, vulnerable persons must be compensated for their time. That is part of justice; in other words, you cannot use their time without their agreement; that will be exploitation. You have to do a lot of things to make sure that the research is done in a proper manner, and you must inform them how long the research will be. Thus, you must inform them about the benefit, you must inform them about the risk. If there is no benefit or if the risk is minimal, you must inform them. Also, you must protect their privacy, maintain the confidentiality of the participants. All these are the key elements that are necessary for the project and for the investigator. So, to meet all these criteria, you have to make sure that you have a comprehensive informed consent document, a detailed protocol that is approved by the appropriate ethics committee.

South African common law also stipulates the necessary requirement for informed consent to be valid, and RM74 highlights this:

There are a number of things according to the common law. Informed consent means you must have knowledge of what you are consenting to; you must appreciate the consequences of what you are consenting to, you must agree to them not being offered pervasive incentive or anything, it must be free, voluntary, and you must consent to all the consequences. OK, that's what the common law says.

In addition, RF46 presented criteria for informed consent set out by the South African National Health Act [63]:

The National Health Act also gives you criteria for informed consent. They say that the participant needs to be given all of the different options, you've got to give, first of all, be told what to use, what they're going to do with you. They have to give you all the different options, but we can do this, that, or whatever it is. You got to then tell them the consequences of the research and so on as well.

The participant went on to point out the need to inform the person that informed consent is being administered to, as it contributes to their understanding and making an informed decision:

They've got to get enough information given in a language they can understand at a level they can understand so if they're not very educated, you've got to talk very simply, so they understand and in the language which they want. (RF46)

The above is for the general consent process. However, when it comes to extreme cases like clinical trials for new drugs, the process is different. RF46 went on to say that in such cases, research participants may need to be taken care of for the rest of their life:

When you're dealing with research, of course, you've got to have a situation where not only do they consent to the procedures and its consequences, but they also need to know what's going to happen once the research finishes, because if you put me on medication and the trial is over, then you withdraw the medication, what's going to happen to me? And so increasingly, as you probably know, ethical committees have to answer to that, and they say you must carry on with the treatment afterward.

Thus, as postulated by most of the participants, informed consent is an essential aspect when it comes to ethical research. However, applying informed consent in Africa and the criteria mentioned above have intrinsic limitations, which we explore in the section below.

### Respondents’ perception of informed consent in Africa

Informed consent is difficult to define in an African context. This is because informed consent is rooted in the Western liberal tradition of individuality. As RM56 puts it:

Informed consent in Africa is a little bit complicated because informed consent in its original idea is derived from a Western conception of libertarian rights. In other words, individual rights which don't really translate to African cultural norm, if you look at it from the point of view of Ubuntu.

To further emphasise this, RM37 states that:

The issue of informed consent and whatever goes with it. It's, it's, it's, it's, it's ancient, its taboo. It's transplanted its ideas or ideologies that are transplanted into our communities we don't have we don't have that, and I do not recall at any point in our lives as black people where we went out, and *toyi toyi* [protest] to say that we want to be autonomous as such.

The traditional African values and norms of behaviour do not advocate for an autonomous existence. Africans have always existed as communities, and this is one of the key common concepts around African communities. In line with this view, RM56 states:

The South African concept of Ubuntu because Ubuntu is not universal, but it gives an idea of the African communitarianism, and this and the concept of ancestors, witchcraft, and relational autonomy is common in most African communities, because if you go to places like Ghana or other African countries you have to get the interest of the community and the interest of the community sometimes overrides the interest of the individual. So, when you compare to the Western concept of informed consent, whereby the rights of the individual always override the right of the community, so there is a conflict there, or there is a difference.

This research study discovered that the current informed consent process might not be directly applicable in Africa owing to limitations ranging from fundamental differences in understanding the autonomy principle to vulnerability and lack of Western education. These are discussed in more detail below.

### Participants’ perception of the application of the principle of respect for autonomy

Applying the principle of respect for autonomy in Africa has been only partially successful, which affects the direct application of the Western informed consent process, because African values and belief systems advocate for communitarianism. Therefore, something that is intrinsically autonomous may not flourish in Africa. This became clearer when RF53 shared her experience of conducting biomedical research in a rural community in the Northern KwaZulu-Natal province of South Africa. She discovered that implementing autonomy and privacy in African communities is very difficult:

For me to talk to the mother and the child, the granny and the father must give me permission. It means now, they are the ones who are allowing that person, so that person is not, there is no autonomy in her because she is not allowed to decide whether she wants it or not. She must first get consent from these two other people or the mother-in-law, must say yes or no or even father-in-law. You see, so that her autonomy is affected. She cannot voluntarily say no I am going to take part. She has got to wait for husband or *gogo* [grandmother] or mother-in-law, you know.

This makes it clear that African research participants are not necessarily going to think as individuals, but rather think as members of a group firmly vested in other members' existence. According to RM56, this is the primary obstacle when it comes to applying the conceptual understanding of autonomy:

The primary obstacle in translating informed consent as it is in the Western construct to the African construct is that the people will not even understand the concept of autonomy because they don't think as individuals.

This mindset, according to RM57, makes medical practice challenging, because when people come to the hospital they often do not come alone. The whole family, including the extended family, brings them in. However, even if they come in alone, he notes that you have to involve the whole family in whatever you do, and this sometimes affects medical interventions. This observation appears consistent with the ethics of care and relational autonomy in African communities, as described by Peter Osuji [31]. In this case, RM57 stated:

In African settings, people come as families. They are brought in by families, and even if they do not come with families, whatever you do to them it affects their families, and in terms of medical intervention, it creates a bit of a challenge because you can sit one on one with the person whom you believe to be autonomous and everything. Then you discuss all the issues that are on the table as far as their conditions are concerned, but the very same person – sometimes you can have a 57-year-old sitting in front of you; your sense is that you can never be more mature than this person, they are as mature as it can get. But the person will tell you that before this intervention, I need to go home and speak to my mother.

Similarly, RM37 shared his experience of working in a community hospital where a patient had developed multidrug-resistant tuberculosis. He acknowledged that in the rural community, the culture of *Ubuntu* was still intact, and the ideology that an individual is part of the community is still at play. In some cases, he had to wait for the patient to go home and tell the people there what is going to happen to him, and RM37 respected that because he knew that after everything, the patient will be sent back to his family where he is a brother, father, uncle, and son. He stated:

I worked in a remote area where the culture is still intact; you are not an individual; you are part of the community. When I see you, I see you as the representative of the community or of even the family. Like in this instance, this man has spoken to me about the fact that he is married, and he also has to speak to his mother before he can leave, and that also meant to us that whatever we do to this person, it means they might also be affected. So, whatever we do with him, we also now have to go outside and go communicate with the family, and we kept him for about two months.

Some participants asserted that the successful application of the principle of autonomy would be impossible, unless one dealt with the paternalism that has been at play in South Africa for years. RM37 stated:

From my experience, I will say that except we are talking about the autonomy against paternalism, that I will advocate for. However, in most cases the person is the person with their community, the autonomy that needs to be entrenched is the one that fights paternalism. This is because the one that fights paternalism is the one where this person has to make a decision about whether he is informed by the decision of his family or not. But I myself as a practitioner must not be on his neck as to what he needs or he does not need, because that's where we come from as South Africans in terms of the history where people decided for us. That's why I am saying some of these issues, they don't come from a vacuum, they come from our terrible past, and how we implement them is another issue. So, if autonomy means taking away paternalism from the practitioner, then by all means; but if the practitioner insists on you being autonomous on your family, but when he cuts off both of your legs, he sends you back to your family, then there is a problem.

Here dealing with the individual as an individual might cause conflict between your practice and the community. RF72 stated:

Applying autonomy was very, very difficult; at a point, we even had to break the rules so we could get the work done and make sure that the community was cared for. We couldn't worry about the individual; we had to worry about the community.

The community/family structure contributes significantly to failure in applying the principle of autonomy in Africa. One of the participants shared her experience during her PhD research study, where she had to go through many processes before speaking with her research participants:

Firstly, as we enter each homestead there is the head of the homestead; you must ask permission from that head. My focus was the mother and the child, but there is the father or the granny. I have to ask permission of the head to find is there a child who is less than two years. They say yes. I say, can I see the mother? They say what you need her for. I ask permission from the head of the homestead, and then the head will allow me, and then in the case where the mother is a teenager, now I need permission from the mother of that young mother. So, it is just, and when I was interviewing because the family sees a stranger, it is a homestead, there are about maybe five structures in one house. It's a homestead. So, we all come here; it's not just me and you, everybody comes here to listen to me, and implementing privacy, that is difficult. You try to tell them, no no, no, I just need this one, but they want to see what you are going to do. (RF53)

This can be attributed to the idea that the communal mindset is deeply embedded in the African thought process; thus, to suddenly think as individuals will be difficult. The participant went on to say that the process would have been more tedious if her research assistant had not understood the local dialect of the people. Thus, her experience and that of other researchers made language one of the limitations that came up repeatedly in this study. This is discussed in detail below.

### Respondents’ perspective on the impact of education on the informed consent process

In addition to the language of the informed consent document, the lack of education among some potential research participants is problematic when obtaining informed consent. Education contributes significantly to one's ability to comprehend the informed consent process and document. Some of the participants asserted that they experienced that the more educated the potential participant is, the more the criteria/implementation of informed consent are understood. Participant RM34 puts this clearly:

Among the rich Africans, there is a level of comprehensibility in terms of informed consent because they are more educated in the Western educational system, and they have a reasonable form of income, they know the consumer rights, and they can ask questions because they have been educated.

However, as a lack of education and poverty are still widespread among Africans who are resident in rural areas, this creates a vulnerability in these communities, as noted by RM56:

The African people that are consenting; the majority of them are not educated, and this is a vulnerability because it makes them vulnerable.

This leaves most Africans in a position in which they have to consent to something that they do not fully understand. This also leads them sometimes to agree simply because the person presenting the research looks educated and sounds like he or she has their best interests at heart. Hence, they go in with the mindset that there is some form of benefit without, weighing up the risks and benefits properly. Most often this desperate move is attributed to poverty; thus, poverty as a limitation will be discussed below.

### Respondents’ perspectives on the impact of poverty on the informed consent process

This limitation during biomedical research has a connection to education. Most Africans lack Western education because they do not have the necessary facilities and financial structure to afford education. This has led to the contextual understanding that African people are generally impoverished, which is an understanding and a reality. RM56 shared the result of a study he conducted in this area:

We conducted a study in South Africa, and we found out that almost 65% of the general population of South Africans that were going to public hospitals have no jobs nor a form of income, including grants.

The above statistics mean that out of every 100 people who go to a public hospital, 65 have no income. This it is not just limited to South Africa and is the reality of most African people, greatly contributing to their vulnerability. Poverty and lack of hope expose most African people to the risk of agreeing to participate in a study even though they are not aware of the dangers, just because there are incentives. Participant RM56 stated:

Poverty makes them vulnerable, especially in the context of research [where] any offer, whether its medication, taxi fare, money, are liable to induce them to agree because they are already in a very, very desperate situation.

Anything that the researcher offers to them—even if it is treatment, which is not good enough motivation to get them to accept—they will accept because there is no alternative. This is called therapeutic misconception, and occurs even during clinical trials [64, 65]. There are situations where people will agree to participate in a trial only because they are going to get some medicine, even though this may or may not be useful to them, because there is a 50% chance that they will receive it. The same happens with money that is paid in compensation for transportation or time. People enrol in research just to get that money, because they do not have any other options for income, so in this context, the consent is not informed consent, but it is induced consent. Participant RM56 went further by stating:

There is a lot of evidence of young people enrolling in research in Africa just because there is money that induces them to participate, and it has to do with the fact that most Africans are generally poor.

This is where values and ethical codes need to be implemented and a support system built to ensure that Africans are not exploited as research objects. Thus, implementing African values and belief systems that advocate for a relational consent process becomes necessary and almost crucial.

## Discussion

### Informed consent and its limitations

This study was designed to interrogate the normative ethical principle of respect for autonomy [3, 21], actualised during biomedical research and clinical practice through the doctrine of informed consent [16, 21, 22, 64]. We wanted to see if we could implement via the empirical bioethics approach in African communities dominated by a culture of communitarianism and relational autonomy; this involves combining normative ethical analysis with empirical data, to arrive at results that would not be possible otherwise [16, 57, 58, 64]. According to Mertz and others [58], ‘empirical ethics’ involves “normatively oriented medical ethical research that directly integrates empirical research” [58]. In other words, empirical bioethics combines both empirical research with normative ethical analysis and tries to integrate both elements to produce new knowledge which might not have been possible otherwise [16, 57, 58, 64]. Here we used qualitative data derived from in-depth interviews with biomedical researchers working in Africa, with detailed analysis of the literature about principlism and respect for autonomy [3] as well as Ross's prima facie duties [34, 35], together with the African communitarian moral philosophy of *Ubuntu* or *Ukama* [10, 23, 29, 32, 37–40], to evaluate the practice of informed consent during biomedical research in South Africa.

This study supports the prevailing view that informed consent is the central defining feature of contemporary ethical biomedical research and clinical practice for researchers and clinicians, even in Africa, consistent with Manson and O'Neil's observation [66] that informed consent is a central concept in contemporary bioethics. It is widely seen as fundamental to ethical conduct of biomedical research in humans [22, 30].

Furthermore, we found that biomedical researchers working in South Africa perceived informed consent as a process that research participants and patients must follow to make research valid. The legal and ethical requirements for the informed consent process in South Africa stipulate that informed consent must involve full disclosure of information [16, 63, 64, 67]. There must be complete comprehension of the information disclosed to research participants or subjects. Further, this consent must be voluntary; that is, the individual must be free to agree or disagree on whether to participate in the research study. It also means that this has to occur in the absence of coercion or undue influence. Moreover, human subjects must not consent out of fear or any other reason whereby they cannot decide based on their own free will [16, 22, 64].

There must also be justice; if individuals have to spend their time and effort to get involved in a research study or project, they must be compensated. Even if they happen to drop out of the research study, vulnerable participants should be compensated for their time. One cannot use their time without their agreement, as that would be equivalent to exploitation. As a researcher, one must disclose all of the benefits that may accrue from participating in the research study; further, one must minimise the risks of research as much as possible. The rights to privacy of the individual participants also have to be protected [16, 22, 64, 67]. All these processes are supported by the South African Constitution [68], common law, the National Health Act 2003 [63], and international ethical guidelines like the Declaration of Helsinki [69].

Guidelines such as those promulgated by the Council of International Organizations of Medical Sciences (CIOMS, 2002) [70], the Nuffield Council on Bioethics (2002) [71], and the National Bioethics Advisory Commission (2001) [72], reinforce commitments that transcend cross-cultural differences, by mandating that the same standards should apply to research participants from both resource-poor and industrialised countries [30, 69–73]. For example, research participants in any cultural setting should provide individual voluntary consent, and studies that could not be conducted in an industrialised country should generally not be implemented in a developing country [72, 73]. These advocates applying the same ethical codes regarding informed consent and guidelines across all cultures [22, 30]. The purpose of such declarations was to reduce exploitation as much as possible, especially among vulnerable communities [73]. Nevertheless, during this study, we discovered that balancing universal and local standards for ethical conduct in biomedical research is challenging, especially when investigators/researchers confront the practical constraints of implementing a study in areas where traditional customs may conflict with international ethical guidelines. This was perceived as a problem in research conducted in South Africa by the respondents in this study, and in studies conducted in other rural communities in Africa [74, 75]. In addition, some participants in this study revealed aspects of ethics of care and relational autonomy which could impact autonomy and the informed consent process in the African setting, as reported by other authors [31].

Furthermore, some respondents in this study highlighted the importance of language and education in informed consent and biomedical research in African communities. Language and education may present a barrier to understanding the informed consent process, as reported by previous studies from South Africa [16, 64, 76]. This consideration prompted the South African National Health Act [63] to require that HCPs obtaining informed consent in South Africa consider healthcare users' language and literacy levels [16, 64, 67, 76–78].

Africa is considered one of the impoverished continents and bears a disproportionate burden of disease morbidity and mortality due to a lack of adequate healthcare resources. It is also struggling to deal with the conflict between general ethical principles and traditional values and norms of behaviour [5, 24, 25, 28, 41, 42, 49]. It is challenging to accomplish the application of general ethical principles underlying guidelines for research conduct, without knowing the cultural context within which a study will occur. Since informed consent is the most important ethical principle for moral conduct of research, it cannot easily be globalised because it is culture dependent. Kuper (1999) states that "Anthropologists have described culture as a symbolic system representing ideas, values, cosmology, morality, and aesthetics, shared by individuals and groups" [79], and this definition goes against the idea of developing a general ethical principle, because 'culture' is particular to a group. Thus, the principles that guide the informed consent process must be flexible in order to fit in with every culture.

However, the principles advocated by most general ethical guidelines are not very flexible, as they are dependent on the Western-European libertarian tradition. Therefore, they emphasise individual autonomy and privacy, which is likely to fail if applied without cultural modifications to traditional societies like those in Africa. This observation is consistent with the arguments of other researchers working on informed consent in Africa [11]. However, one cannot speak about African culture as if it is a homogenous phenomenon. While affirming the diversity among African cultures and its ability to respond to contemporary social and political realities, this study supports Peter Kasenene's position that "despite variety, there is a common ‘Africanness’ about the culture and worldview of Africans" [80]. Some commonalities are shared by indigenous African societies, such as a belief in ancestors, understanding an individual as communally constituted, and a relational worldview [5, 23–27, 81]. One may argue that these commonalities should be the foundational basis for a discourse on African bioethics [28]. Along this line of thought, Akin Makinde argues that:

Theories and practices of medicine have a cultural basis, and it is because of this notion of social embeddedness that African medical practices are inextricable from African culture and belief systems: From this point of view, the concept of illness, diagnosis, treatment, life, and death, must also have a cultural dimension [82]

Makinde's argument has also been echoed by Sindiga, Nyaigotti-Chacha, and Kanunah, who pointed out that:

Each cultural group handles its medical problems in a particular way and has its worldview, traditions, values, and institutions, which have developed over time to address disease and illness. Each culture has its disease etiologies, medical terminologies, classifications, medical practitioners, and a whole range of pharmacopeia. [83]

Sindiga et al.’s observations imply that one cannot postulate, with logical impunity, healthcare practice or ethical methodology as engendering eternal truths that are applicable everywhere, regardless of cultural context. These authors’ analysis established the dichotomy between the Western practice of medicine and biomedical research. Western medicine tends to see disease in terms of the functioning of the body, while Africans may understand diseases in terms of a causal relationship between the "visible" and "invisible world" [83]. In the same vein, Gloria Waite has suggested that an "African understanding of disease should be seen as a medico-religious in contrast to a biotechnical medical system" [84]. Shutte further argues that "a healthcare practice that is purely scientific in its conceptualization and treatment of disease would inevitably fail to embrace the spiritual dimension of human sickness" [85]. Within the traditional African context, such healthcare practice is construed as an exercise in dehumanisation. With its strong emphasis on the idea of the human body's dignity, African bioethics may view Western medical practices as problematic because the human body is treated in such a manner as to regard the person as insentient. In an African cultural context, where a human being is viewed holistically, healthcare practice that emphasises merely repairing human organs is inadequate, because it cannot view disease and causation comprehensively. From this perspective, Janzen states that:

One could argue that this is because of the African holistic view of healing; that is partly why many Africans have resorted to complement Western medicine with that which is provided by African traditional medicine. [86]

One could argue that the practice of complementing Western medicine with African traditional medicine creates room for the implementation of everything, including informed consent processes, which are ontologically Western. However, none of the participants in our study fully agreed with the above arguments. Some point out that with exposure to Western forms of education one may begin to think individually, which creates room for understanding informed consent in an individualistic way [77]. However, with the whole movement towards decolonialization in Africa, it is arguable that Africa's educational systems are beginning to take into consideration key traditional and African cultural values. Therefore, education may not fully resolve the existing conflict between informed consent and conventional African cultural values and behavioural norms [24, 26, 27, 77]. This ever-growing ethical or moral dilemma has led to the emergence of indigenous populations developing their own codes of ethics, such as the San people of Southern Africa [1, 2, 87, 88].

### An alternative approach

This study suggests the need for an alternative approach to the application of informed consent in Southern Africa. The arguments below will combine the findings from the empirical research with existing normative ethics to argue for introducing ethical pluralism in bioethics, while suggesting an alternative approach to ethical decision-making in Africa. Current decision making in bioethics seems to adopt a bottom-up approach to moral reasoning [43, 44]. Since the traditional monistic ethical theories, like consequentialism, utilitarianism, and deontological ethics, appeal to an abstract universal notion of personhood, they could be used as a means of intellectual and cultural imperialism [52, 89, 90].

However, bioethics deals with real people in particular situations: specific persons in specific contexts or contextualized persons. Bioethics is also arguably rooted in culture because there might be no bioethics if there were no cultural practices and way of life [13, 28, 49–52]. Thus, the four assumed globally accepted ethical principles—respect for autonomy, beneficence, non-maleficence, and justice, formally postulated by Beauchamp and Childress since 1989 [3]—address biomedical issues from a Western-European moral and intellectual perspective, and one can posit that the Western-European worldview is different from the African worldview [3–9, 24–29]. In reality, biomedical ethics is assumed to be culture-free and applicable across cultures, despite obvious evidence that its elements and principles are derived from a specific culture and moral tradition [13, 28].

In this study we opted out of a monistic approach in preference of a pluralistic one due to its compatibility with the idea of multiculturalism. As Kevin and Wildes argue, culture has a deep relationship with morality [51]. The concept of multiculturalism links directly to why morality should be pluralistic and account for a diversity of beliefs and value systems [4–9, 50–52]. One can assert that morality encompasses moral practice, which is embedded in culture, which we see as a way of life. Ethics seeks to examine those practices and ethical systems that are embedded in cultural practices; advocating for a multicultural society will be synonymous with supporting a morally pluralistic society [50]. As seen from the above report, there are conflicts and ongoing ethical and moral dilemmas. The differences in cultural and moral values and seemingly intractable problems within the traditional ethical theories indicate the need to look for a principled alternative approach, which is vital to this study. Adopting Ross's model of moral reasoning as a lens, one may argue that it is possible to develop suitable methods to deal with potential conflicts that arise when applying informed consent in Africa, taking into consideration African cultural beliefs and norms of behavior, without undermining traditional African values and belief systems. The central notion of this alternative approach uses Ross's prima facie duties [34, 35], which are different from absolute duties which apply in all circumstances or conditional duties as illustrated by Beauchamp and Childress [3]. We preferred Ross's model because the prima facie duties [35] stem from relationships. Based on the analogy previously illustrated in this article, it is clear that the African notion of personhood is relational. Although Ross's model is Western in origin, it gives credit to relationships, obligations, and responsibility, like the African notion of *Ubuntu*/*Botho*, which advocates for just relationships, mutual interdependence, and humanity [32, 39, 40]. As aptly demonstrated by a landmark judgment of the South African Constitutional Court in *Dikoko v Mokh*a*tla 2006* [40], where a claimant sought reparations in the form of substantial monetary damages from a defendant in a defamation case, the Court argued that *Ubuntu* emphasises restorative justice rather than retributive justice, because restorative justice concentrates more on healing the victims. In her judgment, Justice Mogkoro contended that South African courts should focus on rebuilding relationships between parties, rather than punishing a defendant by awarding heavy fines when deciding defamation cases, since this results in the breakdown of interpersonal relationships. The Court argued that “the primary purpose of a compensatory measure is to restore the plaintiff's dignity who has suffered the damage and not to punish a defendant” [39, 40]. Justice Mokgoro further opined that:

In our constitutional democracy the basic constitutional value of human dignity relates closely to *Ubuntu or Botho*, an idea based on deep respect for the humanity of another […] A remedy based on the idea of *Ubuntu or Botho* could go much further in restoring human dignity than an imposed monetary award in which the size of the victory is measured by the quantum ordered and the parties are further estranged rather than brought together by the legal process. It could indeed give better appreciation and sensitise a defendant as to the hurtful impact of his or her unlawful actions, similar to the emerging idea of restorative justice in our sentencing laws. The focus on monetary compensation diverts attention from two considerations that should be basic to defamation law. The first is that the reparation sought is essentially for injury to one’s honour, dignity, and reputation, and not to one’s pocket. The second is that courts should attempt, wherever feasible, to re-establish a dignified and respectful relationship between the parties. Because an apology serves to recognise the human dignity of the plaintiff, thus acknowledging, in the true sense of *Ubuntu*, his or her inner humanity, the resultant harmony would serve the good of both the plaintiff and the defendant […] The goal should be to knit together shattered relationships in the community and encourage across-the-board respect for the basic norms of human and social interdependence. (*Dikoko v Mokhatla 2006*, paras 68–69) [40].

It is also important to note that Ross's model is not limited to a micro-sociological level; it also calls for issues such as reparations, which can translate to restorative justice at the societal, legal, and policy levels, similar to the *Ubuntu* philosophy, as argued by other commentators [23, 39].

Therefore, based on the above arguments, we suggest that Ross's model seems to be a more suitable model to follow in the African context, because of its emphasis on interpersonal relationships and restorative justice, such as reparations, which appear to be more consistent with the African philosophy of *Ubuntu.* Thus, it may be necessary for political and legal institutions in Africa to undertake reforms to make it possible to apply forms of consent and respect for autonomy that are adapted to Africa's socio-cultural and philosophical contexts. This would include involving family or community members in the consent process during biomedical research [74, 75], or applying the African ethic of *Ubuntu/Botho,* which emphasizes harmony and dignity in human interactions [32, 37–40].

Furthermore, similar to previous reports [16, 64, 76–78], this study noted that the poor educational background in African communities plays a part in comprehending the informed consent process, especially if not administered in a language that the participants understand. Thus, we recommend that African bioethics and biomedical research conducted within African communities consider group consent that aligns with people's literacy, cultural values, and interpersonal relationships.

## Limitations of this study

Evidentiary data on the practical differences and conflicts arising during the experimental process of applying the principle of respect for autonomy and informed consent in some population groups and research participants in Southern Africa would further emphasise this study's claims. Nevertheless, we have bolstered our arguments with reports from other studies in Africa [31–33], including from Ghana [74] and Kenya [75], which support some of this study's findings. Furthermore, this study did not gather the point of view of the persons receiving the consent documents and their relatives, who may be considered co-actors in the construction of relational autonomy in the biomedical researchers' practice. However, this work documented qualitative data and other published literature to address a research problem. The unavailability of such experimental or alternative data does not invalidate the claims that arose from this study. Future studies may be conducted to further validate the observations from this study, which may be limited by time, scope, and location.

## Conclusions

Analysis of empirical data and normative ethical analysis from this study reveals an apparent tension between the Western and African understanding and practice of informed consent and the ethical principle of respect for autonomy during biomedical research in the South African setting. In that case, one can suggest that the Western, libertarian rights-derived informed consent processes may not strictly apply in the African context. However, this does not mean that there is no way it could be adapted to suit the situation in Africa, and it does not also mean that we do not need it at all. However, during this study, it was apparent that researchers must consider the socio-economic status, literacy level, environment, spirituality, and culture of local peoples when dealing with African communities. Similar to what the San peoples of Southern African are advocating for in their code of ethics. When they argue for respect, honesty, truthfulness, and an appreciation that a person is not a person on their own, based on African belief systems, that an individual exists through the community. By default, the person becomes part of a community and whatever happens to the person directly affects the community.

Thus, to extrapolate from Ross, intuitively, a researcher conducting biomedical research in Africa ought to know that what is paramount should be that the way informed consent is practiced in the West is different from how it should be conducted in Africa. It should come at first sight to the researcher that informed consent in Africa should take all the traditional values and belief systems of Africans into account. They should be aware that Africans act as a group, and thus group survival and interests may supersede individual rights. Therefore, while it is possible to implement informed consent in Africa, this implementation has to consider the concept of communitarianism that governs Africans' worldview.

## Supplementary Information

**Additional file 1.** Interview guide. Original interview guide used for in-depth interviews.

**Additional file 2.** COREQ checklist.

**Additional file 3.** Informed consent documents for participants.

## Data Availability

The datasets generated and analyzed during the current study are available from the UKZN ResearchSpace repository, https://researchspace.ukzn.ac.za/handle/10413/16425. However, any additional information analyzed during the present study is available from the corresponding author [SCC] on reasonable request.

## Abbreviations

ACERP: Asian Conference on Ethics Religion and Philosophy
CIOMS: Council of International Organizations of Medical Sciences
HCP: Healthcare Professional
HREC: Humanities Research Ethics Committee
IAFOR: The International Academic Forum
NBAC: National Bioethics Advisory Commission
PI: Principal investigator
SJTI: St Joseph’s Theological Institute, South Africa
UKZN: University of KwaZulu-Natal
USA: United States of America
WMA: World Medical Association
WHO: World Health Organization
