# Supplementary material for: South African traditional values and beliefs regarding informed consent and limitations of the principle of respect for autonomy in African communities: a cross-cultural qualitative study
Source: BMC Med Ethics. 2021 Aug 14;22:111. doi: 10.1186/s12910-021-00678-4 (PMC8364064; doi:10.1186/s12910-021-00678-4)
Supplement: Supplementary file 1 — Additional file 1. Interview guide. Original interview guide used for in-depth interviews. [file 12910_2021_678_MOESM1_ESM.docx]

## Original Interview guide

Thank you very much for agreeing to speak with me about your experience of medical research in African. I am Francis Fabian Akpa-Inyang working on a research titled; Southern African traditional values and belief systems and informed consent process in biomedical ethics: perceptions of the San code of ethics. Before we start I will like to know your gender, age if you are comfortable, race and your research specialty.

1. What are the important aspects of an ethical research project for medical researchers
2. What are the strategies you adopt as a principle investigator to meet these criteria?
3. Are there any limitations in the application of these criteria to the general population of Africa?
4. What is your opinion on informed consent in Africa?
5. How successful is the application of the principle of autonomy in the general population of Africa?
6. Are you aware that the San people designed a new code of ethics? (if no I will explain)
7. What do you think about the San code of ethics?
8. What are the possible contributions of the San’s code of ethics to medical research and practice in Africa?
9. Looking at Ross’ theory of *Prima Facie* duties, do you think that adopting a principle that is more cultural sensitive will engender the relationship between researcher and research participants?
10. Is it possible to reconcile the concept of informed consent with the traditional African values and norms of behaviour taking the San code of ethics as a case?
11. What is the suitable method for the recognition of arising moral conflicts and controversies and their resolution with respect to traditional values and beliefs?
