# Supplementary material for: South African traditional values and beliefs regarding informed consent and limitations of the principle of respect for autonomy in African communities: a cross-cultural qualitative study
Source: BMC Med Ethics. 2021 Aug 14;22:111. doi: 10.1186/s12910-021-00678-4 (PMC8364064; doi:10.1186/s12910-021-00678-4)
Supplement: Supplementary file 2 — Additional file 2. COREQ checklist. [file 12910_2021_678_MOESM2_ESM.docx]

**COREQ (Consolidated criteria for Reporting Qualitative research) Checklist**

| Topic | Item No. | Guide Questions/Description | Reported on page No. | |
| --- | --- | --- | --- | --- |
| \| **Domain 1: Research team and reflexivity** \| \| --- \| | | | | |
| \| *Personal characteristics* \| \| --- \| | | | | |
| Interviewer/facilitator | 1 | Which author/s conducted the interview or focus group? **(FA-I)** | 14 | |
| \| Credentials \| \| --- \| | 2 | What were the researcher’s credentials?  **BA Philosophy, BA (Hons) Applied Ethics** | 1,37 | |
| \| Occupation \| \| --- \| | 3 | What was their occupation at the time of the study?  **Masters Candidate in Population Studies** | 37 | |
| \| Gender \| \| --- \| | 4 | Was the researcher male or female?  **Male** | n/a | |
| \| Experience and training \| \| --- \| | 5 | What experience or training did the researcher have? *The researcher had experience in writing qualitative research at honours level and had completed academic modules on qualitative and quantitative research methods. Entire study was supervised by a PhD level researcher* | 15, 37  Also see ref. no. 36 | |
| \| *Relationship with participants* \| \| --- \| | | | | |
| \| Relationship established \| \| --- \| | 6 | Was a relationship established prior to study commencement? | 13-15 | |
| \| Participant knowledge of the interviewer \| \| --- \| | 7 | \| What did the participants know about the researcher? e.g. personal goals, reasons for doing the research \| \| --- \| | 13-15 | |
| \| Interviewer characteristics \| \| --- \| | 8 | \| What characteristics were reported about the inter viewer/facilitator? e.g. Bias, assumptions, reasons and interests in the research topic \| \| --- \| | 15 | |
| \| **Domain 2: Study design** \| \| --- \| | | | | |
| \| *Theoretical framework* \| \| --- \| | | | | |
| \| Methodological orientation and Theory \| \| --- \| | 9 | \| What methodological orientation was stated to underpin the study? **Empirical bioethics (using qualitative data), discourse analysis- (normative ethics, ethical pluralism, and duty-based ethics*;* as well as content analysis** \| \| --- \| | 12-15 | |
| \| *Participant selection* \| \| --- \| | | | |  |
| \| Sampling \| \| --- \| | 10 | \| How were participants selected? e.g. **snowball and purposive sampling** \| \| --- \| | 13-14 | |
| \| Method of approach \| \| --- \| | 11 | \| How were participants approached? e.g. **face-to-face and by email** \| \| --- \| | 13-15 | |
| \| Sample size \| \| --- \| | 12 | \| How many participants were in the study? \| \| --- \| \| **12** \| | 13-14 | |
| \| Non-participation \| \| --- \| | 13 | \| How many people refused to participate or dropped out? **3** Reasons? **Time constraints** \| \| --- \| | 15 | |
| \| s*etting* \| \| --- \| | | | | |
| \| Setting of data collection \| \| --- \| | 14 | \| Where was the data collected?  **workplace** \| \| --- \| | 14 | |
| \| Presence of non-participants \| \| --- \| | 15 | \| Was anyone else present besides the participants and researchers? **NO** \| \| --- \| | 14 | |
| \| Description of sample \| \| --- \| | 16 | \| What are the important characteristics of the sample? e.g. demographic data, date \| \| --- \| | 13-16 | |
| \| *Data collection* \| \| --- \| | | | | |
| \| Interview guide \| \| --- \| | 17 | \| Were questions, prompts, guides provided by the authors? **Yes, *an original interview guide was used, developed from literature review* (see additional file 1 )** Was it pilot tested? No, it *was not pilot tested* \| \| --- \| | 44 | |
| \| Repeat interviews \| \| --- \| | 18 | \| Were repeat inter views carried out? If yes, how many? **No,** *as the audio recordings were clear enough.* \| \| --- \| | 14-26 | |
| \| Audio/visual recording \| \| --- \| | 19 | \| Did the research use audio or visual recording to collect the data? **Yes** \| \| --- \| | 14 | |
| \| Field notes \| \| --- \| | 20 | \| Were field notes made during and/or after the interview ? **Yes** \| \| --- \| | 14 | |
| \| Duration \| \| --- \| | 21 | \| What was the duration of the interviews ? **30-40minutes** \| \| --- \| | 14 | |
| \| Data saturation \| \| --- \| | 22 | \| Was data saturation discussed? \| \| --- \| \| *It was not discussed as the target population was obtained* \| | 14-15 | |
| \| Transcripts returned \| \| --- \| | 23 | \| Were transcripts returned to participants for comment and/or correction? **No,** *as the audio recordings were clear enough.* \| \| --- \| | 14-26 | |
| **Domain 3: analysis and findings** | | | | |
| *Data analysis* | | | | |
| Number of data coders | 24 | How many data coders coded the data? **One,** **(F-AI- the PI),** results and analysis were crosschecked candidate’s dissertation supervisor | 14-15 | |
| Description of the coding tree | 25 | Did authors provide a description of the coding tree? **Yes** | 15 | |
| Derivation of themes | 26 | Were themes identified in advance or derived from the data? Derived from the data | 15-26 | |
| Software | 27 | What software, if applicable, was used to manage the data? **NO** | n/a | |
| Participant checking | 28 | Did participants provide feedback on the findings?  **No,** *as the audio recordings were clear enough.* | 14-26 | |
| \| *Reporting* \| \| --- \| | | | | |
| \| Quotations presented \| \| --- \| | 29 | Were participant quotations presented to illustrate the themes/findings? Was each quotation identified? e.g. participant number **YES** | 15-26 | |
| Data and findings consistent | 30 | Was there consistency between the data presented and the findings? **YES** | 15-26 | |
| Clarity of major themes | 31 | Were major themes clearly presented in the findings? **YES** | 15-26 | |
| Clarity of minor themes | 32 | Is there a description of diverse cases or discussion of minor themes? **YES** | 15-26 | |

Developed from: Tong A, Sainsbury P, Craig J. Consolidated criteria for reporting qualitative research (COREQ): a 32-item checklist for interviews and focus groups. *International Journal for Quality in Health Care*. 2007. Volume 19, Number 6: pp. 349 – 357
