# Supplementary material for: South African traditional values and beliefs regarding informed consent and limitations of the principle of respect for autonomy in African communities: a cross-cultural qualitative study
Source: BMC Med Ethics. 2021 Aug 14;22:111. doi: 10.1186/s12910-021-00678-4 (PMC8364064; doi:10.1186/s12910-021-00678-4)
Supplement: Supplementary file 3 — Additional file 3. Informed consent documents for participants. [file 12910_2021_678_MOESM3_ESM.docx]

1. **UKZN HUMANITIES AND SOCIAL SCIENCES RESEARCH ETHICS COMMITTEE (HSSREC)**

**INFORMED CONSENT**

**Information Sheet and Consent to Participate in Research**

Date: 15 May 2017

Greeting: Dear Researcher,

My name is Francis Fabian Akpa-Inyang a master’s student in The School of Build Environment and Developmental Studies, Department of Population Studies.

Contact: 0604147582

Email: francisediomo@gmail.com

You are being invited to consider participating in a study that involves ‘Southern African traditional values, belief systems, and the informed consent process in biomedical research: Perceptions of the San’s Code of Ethics’. The aim and purpose of this research is

1. To explore the understandings of the nature of the principle of informed consent among biomedical researchers.
2. To explore the possible conflicts that will arise in the application of the principle of medical ethics to the general population in Africa…
3. To determine the reasons and grounds for conflicts and controversies that its application can engender in the context of African traditional value and belief systems.
4. To argue for the use of an alternative approach to explore the concept of informed consent in doctor patient relationship in Africa.

The study is expected to enrol 12 medical researchers in University of KwaZulu-Natal and CAPRISA. It will involve interview sessions. The duration of your participation if you choose to enrol and remain in the study is expected to be 1hour. The study is funded by the department of Population Studies in University of KwaZulu-Natal.

The study do not involve any risks and/or discomforts. The hope is that the study will create a new biomedical principle for medical practitioners and researchers that will be applicable for African people in Africa.

This study has been ethically reviewed and approved by the UKZN Humanities and Social Sciences Research Ethics Committee (approval number_____).

In the event of any problems or concerns/questions you may contact the researcher at

School of Build Environment and Development Studies,

Department of Population Studies,

Howard College Campus,

Shepstone Building Level 7,

Durban

4000

KwaZulu-Natal, South Africa

Cell:+27604147582

Email:francisediomo@gmail.com

or the UKZN Humanities & Social Sciences Research Ethics Committee, contact details as follows:

**HUMANITIES & SOCIAL SCIENCES RESEARCH ETHICS ADMINISTRATION**

Research Office, Westville Campus

Govan Mbeki Building

Private Bag X 54001
Durban
4000

KwaZulu-Natal, SOUTH AFRICA

Tel: 27 31 2604557- Fax: 27 31 2604609

Email: [HSSREC@ukzn.ac.za](mailto:HSSREC@ukzn.ac.za)

Participation in this research is voluntary and participants have the freewill to withdraw participation at any point. In addition, at the event of refusal/withdrawal of participation the participants will not incur penalty or loss of treatment or other benefit to which they are normally entitled. The participant can terminate participation in the study at any time and it will not incur any consequence. The researcher will terminate a participant from the study if and only if the participant is not a medical researcher or practitioner.

There will be no cost incurred by the participant as a result of participation in the study and there will be no incentives or reimbursement for participation in the study.

To protect confidentiality of personal/clinical information, I have decided that the digital recordings and electronic transcripts will be kept in a password protected folder on my personal computer. The hard copies will be locked in a filing cabinet. No identifying information (such as consent forms) will be kept with the digital or hard copies. On completion of the project, the research data (all electronic and hard copies) will be stored in the supervisor’s office for five years after which it will be permanently destroyed.

------------------------------------------------------------------------------------------------------------------

**CONSENT (Edit as required)**

I __________________________________________________ have been informed about the study entitled ‘Southern African traditional values and belief systems and the informed consent process in biomedical research: Perceptions of the San’s Code of Ethics’ by Francis Fabian Akpa-Inyang from the School of Build Environment and Development Studies, department of Population Studies.

I understand the purpose and procedures of the study.

I have been given an opportunity to answer questions about the study and have had answers to my satisfaction.

I declare that my participation in this study is entirely voluntary and that I may withdraw at any time without affecting any of the benefits that I usually am entitled to.

If I have any further questions/concerns or queries related to the study I understand that I may contact the researcher at (provide details).

If I have any questions or concerns about my rights as a study participant, or if I am concerned about an aspect of the study or the researchers then I may contact:

**HUMANITIES & SOCIAL SCIENCES RESEARCH ETHICS ADMINISTRATION**

Research Office, Westville Campus

Govan Mbeki Building

Private Bag X 54001
Durban
4000

KwaZulu-Natal, SOUTH AFRICA

Tel: 27 31 2604557 - Fax: 27 31 2604609

Email: [HSSREC@ukzn.ac.za](mailto:HSSREC@ukzn.ac.za)

**____________________ ____________________**

**Signature of Participant Date**

**____________________ _____________________**

**Signature of Witness Date**

**(Where applicable)**

**____________________ _____________________**

**Signature of Translator Date**

**(Where applicable)**
